# How (much) do flowers vary? Unbalanced disparity among flower functional modules and a mosaic pattern of morphospace occupation in the order Ericales

**DOI:** 10.1098/rspb.2017.0066

**Published:** 2017-04-05

**Authors:** Marion Chartier, Stefan Löfstrand, Maria von Balthazar, Sylvain Gerber, Florian Jabbour, Hervé Sauquet, Jürg Schönenberger

**Affiliations:** 1 Department of Botany and Biodiversity Research, University of Vienna, Rennweg 14, 1030 Vienna, Austria; 2 Department of Ecology, Environment and Plant Sciences, Stockholm University, SE-106 91 Stockholm, Sweden; 3 Muséum national d'Histoire naturelle, Institut de Systématique, Évolution, Biodiversité, UMR 7205 ISYEB MNHN/CNRS/UPMC/EPHE, 57 rue Cuvier, CP 39, 75005 Paris, France; 4 Laboratoire Écologie, Systématique, Évolution, Université Paris-Sud, CNRS UMR 8079, 91405 Orsay, France

**Keywords:** disparity, Ericales, flower morphology, fossils, functional modules, morphospace

## Abstract

The staggering diversity of angiosperms and their flowers has fascinated scientists for centuries. However, the quantitative distribution of floral morphological diversity (disparity) among lineages and the relative contribution of functional modules (perianth, androecium and gynoecium) to total floral disparity have rarely been addressed. Focusing on a major angiosperm order (Ericales), we compiled a dataset of 37 floral traits scored for 381 extant species and nine fossils. We conducted morphospace analyses to explore phylogenetic, temporal and functional patterns of disparity. We found that the floral morphospace is organized as a continuous cloud in which most clades occupy distinct regions in a mosaic pattern, that disparity increases with clade size rather than age, and that fossils fall in a narrow portion of the space. Surprisingly, our study also revealed that among functional modules, it is the androecium that contributes most to total floral disparity in Ericales. We discuss our findings in the light of clade history, selective regimes as well as developmental and functional constraints acting on the evolution of the flower and thereby demonstrate that quantitative analyses such as the ones used here are a powerful tool to gain novel insights into the evolution and diversity of flowers.

## 1. Introduction

Angiosperm evolution has given rise to an overwhelming diversity of floral morphologies adapted to pollination by a multitude of different vectors. This diversity is mirrored in the high variability of breeding systems and reproductive strategies across angiosperms. Hence, it is hypothesized that floral form and function have important effects on diversification [1–4]. There is an extensive body of literature on floral morphology, pertaining both to extant and extinct taxa [5–11]. However, the distribution of flower morphological diversity across major subclades, let alone across the angiosperms as a whole, has rarely been addressed using an explicitly analytical and synthetic approach [12,13]. Such broad-scale analyses of disparity (morphological diversity) have so far been largely restricted to animal groups [14–17].

Morphospace analyses are used to study macro-evolutionary patterns and trends in disparity within and among clades. While disparity analyses are traditionally conducted on large numbers of traits capturing the overall morphology of particular organisms [18–21], some studies have focused on sets of traits of specific functional, developmental or evolutionary significance (e.g. the morphology of animal jaws in relation to feeding behaviour [22–24]). This latter approach also allows us to account for the fact that different traits might evolve with different modes and rates [25] and at different times in the history of clades [26]. Under the assumption that traits/subsets of traits involved in different functions are subject to different evolutionary constraints and selective regimes, one might expect these traits to show different levels of disparity. Studies on significant subsets of organs are thus necessary to clearly characterize the different drivers and causes underlying the disparity exhibited by a clade; such studies provide an alternative and complementary approach to traditional comparative structural analyses.

Most flowers are composed of three main functional modules. From the periphery to the centre, a flower usually comprises one or two sets of sterile organs (perianth), a set of male reproductive organs (androecium) and a set of female reproductive organs (gynoecium; electronic supplementary material, figure S1). In the perianth, sepals commonly protect younger organs during pre-anthetic stages, while petals mainly attract and guide pollinators at anthesis [27]. The function of the androecium is pollen production and presentation, and, more rarely, pollinator attraction. Finally, the main functions of the gynoecium are ovule production, pollen reception and sustenance of pollen tube growth, as well as seed protection and dissemination. The organization and development of these three functional modules are not only the basis of traditional, taxonomic descriptions and comparative analyses of floral structure, but are also the target of modern, molecular developmental (evo-devo) models of floral evolution and development such as the ABCE-model [28,29]. Recent research has also focused on the synorganization of functional units in flowers [11,30,31]. However, the allocation of morphological variation among these three floral components has never been quantified. Here, we tested whether disparity in the flower as a whole is equally reflected in the three functional modules, or whether, by contrast, one part of the flower varies more than the others. At the scale of an entire plant order, where organs can be compared at the organizational and functional level, we expected the perianth to show low variability, owing to the simple structure of petals and sepals. On the other hand, the gynoecium is a very complex structure, achieving numerous functions throughout the flower's life [7,32], and we expected it to show high variability when compared with the rest of the flower.

**Figure 1.**
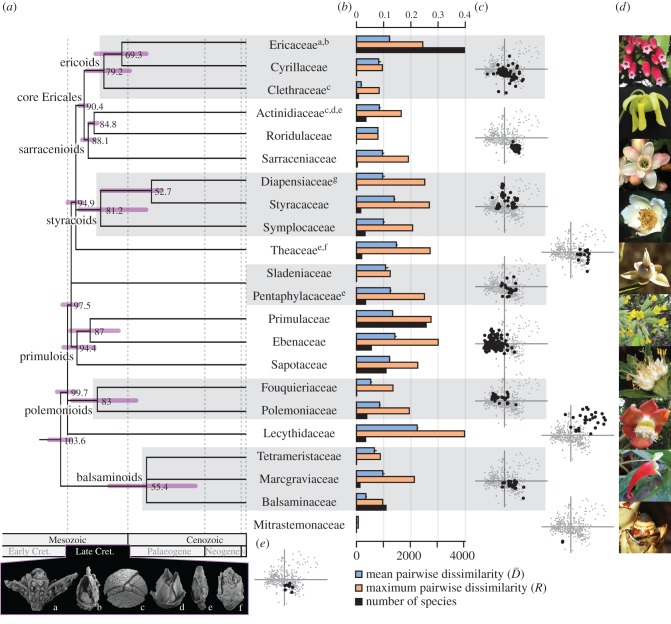
The floral morphospace of Ericales. (*a*) Phylogenetic relationships among ericalean families; dated tree modified from [33], keeping only nodes (with crown ages given in Ma) that are supported in [35]. Pictures of six of the fossil genera included in the analyses: a *Raritaniflora*, b *Paleoenkianthus*, c *Glandulocalyx*, d *Parasaurauia*, e *Paradinandra* and f *Pentapetalum*. Assigned positions (according to original papers) of the fossils are highlighted in (*a*) by superscript letters on the family names. g Proposed positions of *Actinocalyx* (picture not shown). (*b*) Disparity. In blue: mean pairwise dissimilarity; in orange: maximum pairwise dissimilarity (range) rarefied to 10; in black: number of species according to [34]. Error bars are bootstrapped s.e. (*c*) Morphospace representation using principal coordinate analysis. Each graph corresponds to the two-dimensional representation of the space. Black dots: species of highlighted major suprafamilial clades or families; grey dots: all remaining ericalean species. (*d*) Illustration of floral diversity in Ericales: from top to bottom: *Satyria* sp.* (Ericaceae), *Sarracenia flava* (Sarraceniaceae), *Symplocos pendula* (Symplocaceae), *Schima superba* (Theaceae), Anneslea fragrans** (Pentaphylacaceae), Primula officinalis* (Primulaceae), *Cantua quercifolia* (Polemoniaceae), Couroupita guianensis* (Lecythidaceae), *Impatiens paucidentata* (Balsaminaceae), Mitrastemon matudae*** (Mitrastemonaceae). (*e*) Position of the nine fossil species (black dots) in the morphospace (grey dots). Fossil pictures: a republished with permission of The University of Chicago Press from [36]; b, e and f republished with permission of the Botanical Society of America from [37–39]; c republished with permission of Oxford University Press from [40], permission conveyed through Copyright Clearance Center, Inc.; d by P. Herendeen. Photos by *A. Weissenhofer; **T. Rodd; ***D. Breedlove, included with the authorization of D. L. Nickrent. (Online version in colour.)

We addressed these issues in the Ericales, a speciose order of angiosperms nested in the asterid clade of core eudicots. Ericales diverged from their sister group in the Early Cretaceous, *ca* 112 million years ago [33] and encompass 22 families (APG IV [34]; figure 1*a*), 346 genera, and approximately 11 550 species [41] displaying considerable ecological diversity [42]. In many tropical rainforests, ericalean taxa account for up to 10% of the total tree species diversity [43]. The order includes species of great economic importance, such as tea (Theaceae), kiwi (Actinidiaceae), persimmon and ebony (Ebenaceae), Brazil nuts (Lecythidaceae), sapote (Sapotaceae, Ebenaceae) and a variety of ornamental species such as heather and rhododendrons (Ericaceae), and primroses (Primulaceae). Ericales have a worldwide distribution and a considerable diversity in habit, general morphology, method of nutrient uptake, and in particular, floral morphology [42,44]. This diversity is also reflected by the fact that the identification of non-molecular synapomorphies for the order as a whole has been proven difficult, while detailed comparative studies of floral structure have identified series of potential synapomorphies for various suprafamilial clades [45–47]. Importantly, Ericales also have a comparatively rich fossil record with a series of charcoalified flower fossils from the Late Cretaceous, the geologic period during which the angiosperms began to dominate most terrestrial ecosystems [36,38,40]. As the charcoalification process preserves the three-dimensional shape of floral fossils and only leads to moderate alterations at the morphological level (e.g. shrinkage [48]), most of these fossil flowers are extremely well preserved and can be compared directly with their extant relatives [8,48].

For this study, we compiled an extensive dataset of extant and fossil ericalean species and built a floral morphospace based on 37 traits capturing the morphology of their flowers. Our first goal was to quantify patterns of morphospace occupation within and among ericalean families and suprafamilial clades. We tested the hypothesis (i) that various suprafamilial clades do not overlap or only overlap partly in the floral morphospace. This hypothesis derives from the fact that several clades such as, for instance, the ericoid and the primuloid clade, were not considered to be closely related in pre-molecular, largely morphology-based classifications (e.g. [49]), suggesting divergent floral morphologies. At the same time, we hypothesized (ii) that families that are supported as closely related based on comparative floral structure (such as the balsaminoid families or the polemonioid families), will occupy overlapping areas in the morphospace due to their relatively recent common ancestry. We then used our dataset to investigate (iii) whether floral disparity is coupled with clade age and/or species richness. Furthermore, we placed several Cretaceous ericalean fossils in the floral morphospace of extant taxa. Based on their old age and the fact that most of these fossils have been referred to different ericalean lineages, we hypothesized (iv) that they fall in different areas of the total floral morphospace of Ericales. Finally, we compared the disparity of the sterile, male and female parts of the flower, to test the hypothesis (v) that levels of disparity differ according to the biological or ecological function of organ modules, reflecting different evolutionary constraints and selective regimes.

## 2. Material and methods

### (a) Taxon sampling

We sampled 381 species belonging to 275 genera (accepted in [50]). For each family, we sampled at least one species per genus, to ensure that our sample is representative of the families' floral morphological diversity. When there were less than 10 genera in a family (that was the case for 14 families, see electronic supplementary material, figure S2), we sampled at least 10 species whenever possible (electronic supplementary material, figure S2). For the families Ericaceae (126 genera), Primulaceae (68 genera) and Sapotaceae (60 genera), we sampled at least 50 genera, taking care to cover all major clades identified in phylogenetic/taxonomic studies (e.g. [51–53]).

### (b) Character set and character coding

We used original species descriptions or, when available, recent taxonomic revisions, online floras, and other scientific literature, as well as personal observations from living and alcohol collections of the Botanical Garden of the University of Vienna. We scored 37 floral characters describing: general features (e.g. flower size; five characters), the perianth (12 characters), the androecium (13 characters) and the gynoecium (six characters) of the anthetic flower. These characters were chosen for their capacity to characterize the number and position of organs, organ union (organs of the same type), organ fusion (organs of different types) and organ form (for staminodes). Using taxonomic keys as our primary source, we selected all the characters that described the flower and that were applicable throughout the whole order of Ericales.

For species producing unisexual flowers, characters of androecium and gynoecium organs were only scored for functional organs and not for sterile organs, such as staminodes and pistillodes.

Some characters, such as organ number and size, were frequently described as polymorphic in the literature. As the frequencies of these variations were rarely detailed, we choose to code the most common state, when it was documented. For instance, ‘(4-) 5 (-6) petals’ in a description was coded ‘5’ in our dataset. The remaining polymorphic characters (e.g. ‘4–6 petals’) were coded as such, which represents 274 data entries (2.2% of all data entries; electronic supplementary material, table S2). As most of our analyses do not support polymorphisms, we randomly sampled a matrix (for each analysis), in which each of the polymorphic cells was replaced by a value comprised of the cell range (for numerical discrete data) or by one of the possible states (for binary and categorical data). Given the low amount of polymorphic data entries, this matrix sampling had no effect on the statistics calculated nor on data visualization (data not shown).

Data were entered and are stored in the online database PROTEUS [54]. Each data entry, user name, source and putative notes are available in electronic supplementary material, table S2. The detailed description of the characters and character states is given in the electronic supplementary material.

### (c) Dissimilarity matrix

All our analyses were performed using the software R v. 3.0.0 [55]. Scripts are available upon request from M. Chartier and S. Gerber.

We calculated pairwise dissimilarities between taxa using the *mean character difference* (here noted *D*) [56]. Let us have two taxa *A* and *B* described by *N* morphological characters. For a character i, the difference *d_AB_*_i_ between *A* and *B* was calculated in different ways depending on the type of character:

— for numerical characters, *d_AB_*_i_ was calculated as the absolute value of the difference between the values of the character for *A* and *B*, divided by the range of the character in the dataset;
— for ordered categorical characters, *d_AB_*_i_ was calculated as the number of steps between the values of the character for *A* and *B*, divided by the maximum possible step difference for the character in the dataset;
— for binary and unordered categorical characters, *d_AB_*_i_ took the value {1} if *A* and *B* shared the same state, {0} if not;
— if the value of a character was missing for *A* or/and *B*, this character was removed from the calculation of *D. N* was thus reduced to the number *N'* of characters with no missing data for *A* or *B*.

The mean character difference *D_AB_* between taxa *A* and *B* was finally computed as

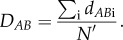
*D* was calculated for each pair of taxa to create the dissimilarity matrix. Note that characters ‘19. Number of stamens’ and ‘36. Number of ovules per carpel’ were log transformed to reduce the weight of extremely high (and rare) values on the analyses, observed in the distributions of these two characters only.

### (d) Morphospace and disparity

To illustrate morphological differences between the 22 Ericales families, we visualized the morphospace of Ericales with a principal coordinate analysis (PCoA; [57]) taking as input the original dissimilarity matrix.

Calculation and analyses of the morphological diversity (disparity) were carried out from the original dissimilarity matrix, and not from the ordination scores. Disparity within each family was calculated as the mean pairwise dissimilarity, i.e. the mean *D* per family (here noted 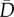; [58]), and as the range (here noted *R*, the maximum value of *D* for a family; [59]). Contrary to 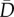, *R* is sensitive to sample size [60]. We thus rarefied *R* to 10 (our minimum sample size whenever possible).

Partial disparity (*PDiv*, the additive contribution of each family to the disparity of the whole order) was calculated following [61]. It is the sum, over each PCoA axis, of the squared Euclidean distances between all taxa from a clade and the centroid of the whole dataset (divided by the total number of species in the dataset).

### (e) Interfamilial comparison

Two groups falling in different parts of the morphospace are morphologically different, whereas they are similar if their distributions in the space overlap. We assessed morphological differences among ericalean families with non-parametric analyses of variance (npMANOVA, sometimes also referred to as PERMANOVA) using the function adonis() from the *vegan* package in R [62]. We used the original dissimilarity matrix as input, and 10 000 permutations to calculate the distribution of a pseudo *F* ratio under the null hypothesis. We used the same analysis as post hoc, with a Bonferroni correction.

### (f) Correlations between disparity and clade size/clade age

Spearman correlation tests were performed to investigate the links between disparity (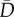 and *R*) and the number of species per family as reported in [41]. The same test was performed to investigate the link between disparity (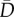 and *R*) and the stem age of families as estimated in [33], for only those nodes that were supported in [35].

### (g) Trait variation

To compare variation among the 37 morphological traits, we averaged *d*_AB_, i.e. the differences between taxa, for each character. The resulting values, here noted *Dchar*, increase with the variation of a character in the dataset.

### (h) Comparison of the disparity of floral functional modules

In our dataset, the perianth morphospace is based on 12 characters, the androecium space on 13 characters, and the gynoecium space on seven characters. Because these three morphospaces differ in character composition and in size, their respective disparities cannot satisfactorily be compared directly. We first investigated if the disparity for each of the functional modules increased with total disparity in Ericales. To do so, we performed a Mantel test, to test for a correlation between the disparity matrices (*D* for each taxa pair) calculated for each of the modules' character sets, respectively, and the disparity matrix calculated for the whole character set. Taxa pairs for which *D* could not be computed for one of the functional modules, due to missing data, were pruned from the matrices before performing the test.

To assess if the perianth, androecium and gynoecium of Ericales are more or less variable than the rest of the flower, we then compared the disparity (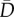) of the whole dataset associated with the perianth (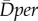), the androecium (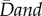), and the gynoecium (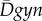) to the distributions of 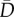 calculated for random character sets of the same sizes (similarly to the method proposed in [63] for assessing the significance of the Escouffier' RV coefficient value). Using the total taxon set, each of these distributions was obtained by calculating 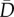 for 1000 matrices of respectively 12 (to be compared with the perianth), 13 (to be compared with the androecium) and seven (to be compared with the gynoecium) characters randomly sampled without replacement in the character set. Perianth, androecium and gynoecium were considered as significantly more (or less) variable than the rest of the flower if 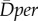, 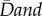 and 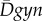 were higher (or lower) than 97.5% of the 1000 randomly sampled 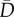 values. We calculated pseudo *p*-values *p* as the proportion of the randomly sampled 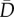 that were higher (lower) than 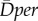, 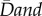 and 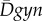. As this is a two-tailed test, the presented values of *p* are corrected (by adding 0.025) to match the usual 0.05 threshold value.

### (i) Incorporation of floral fossils

In addition to the 381 extant species, nine ericalean floral mesofossils from the Cretaceous were added: *Actinocalyx bohrii* (Diapensiaceae; [64]), *Glandulocalyx upatoiensis* (Actinidiaceae or Clethraceae; [40]), *Parasaurauia allonensis* (Actinidiaceae; [65]), *Paleoenkianthus sayrevillensis* (Ericaceae; [37]), *Paradinandra suecica* (Pentaphylacaceae, Theaceae or Actinidiaceae; [38]), *Pentapetalum trifasciculandricus* (Theaceae; [39]), *Raritaniflora glandulosa*, *R. sphaerica* and *R. tomentosa* (Ericales; [36]). Morphospace and partial disparity were recomputed for the dataset including these fossils.

## 3. Results

### (a) The morphospace of Ericales

Our dataset contains 12 512 data entries. In total, 1927 (13.4%) data are missing. The average percentage of missing data is 13.4 ± 10.3 (mean ± s.d.) per taxon and 13.4 ± 12.2 per character.

The floral morphospace of Ericales is organized in a continuous cloud (figure 1*c*; electronic supplementary material, interactive three-dimensional figure S3). The first three principal coordinate axes of the space representation summarized 31.5% of the original variance (15.8%, 8.45% and 7.25% respectively, electronic supplementary material, figure S3), the first two, 24.2% (figure 1*c*). Both three-dimensional and two-dimensional representations gave a fair approximation of the relative dissimilarity among taxa (Pearson's *r* = 0.79, *p* < 0.001 for three axes; Pearson's *r* = 0.71, *p* < 0.001 for two axes). In this space, most of the seven suprafamilial clades, plus the families Theaceae, Lecythidaceae and Mitrastemonaceae, occupy distinct neighbouring regions arranged in a mosaic pattern (PERMANOVA: *F* = 23.24, *r*^2^ = 0.36, *p* < 10^−4^; table 1 and figure 1*c*; electronic supplementary material, figure S3). The only exception is the balsaminoid clade, which does not significantly differ from most of the other suprafamilial clades, mainly because two of its three families, Tetrameristaceae and Marcgraviaceae, overlap with most clades in the order (PERMANOVA: see electronic supplementary material, table S1).

**Table 1.** Post hoc pairwise comparisons (PERMANOVA) based on floral traits among Ericales' supra familial clades. *F* (upper diagonal) and *r*^2^ (lower diagonal) values are given for significantly different comparisons. n.s. = clades that are not significantly different. Overall test: PERMANOVA: *F* = 23.24, *r*^2^ = 0.36, *p* < 10^−4^. Pent + Slad = clade composed of Pentaphylacaceae and Sladeniaceae.

	Balsaminoids	Polemonioids	Primuloids	Pent + Slad	Lecythidaceae	Mitrastemonaceae	Theaceae	Styracoids	Sarracenioids	Ericoids
Balsaminoids		8.306	17.972	n.s.	14.909	n.s.	n.s.	n.s.	n.s.	11.024
Polemonioids	0.145		9.603	11.242	57.521	7.72	20.291	9.03	29.153	12.434
Primuloids	0.105	0.057		22.803	70.461	6.659	27.008	21.137	39.382	55.534
Pent + Slad	n.s.	0.187	0.13		52.154	n.s.	11.085	9.807	12.576	6.831
Lecythidaceae	0.237	0.52	0.31	0.521		n.s.	17.253	56.59	34.234	81.301
Mitrastemonaceae	n.s.	0.216	0.048	n.s.	n.s.		n.s.	n.s.	9.083	9.09
Theaceae	n.s.	0.331	0.157	0.235	0.301	n.s.		16.894	n.s.	19.053
Styracoids	n.s.	0.12	0.111	0.138	0.465	n.s.	0.242		24.212	17.787
Sarracenioids	n.s.	0.373	0.205	0.222	0.416	0.283	n.s.	0.284		20.806
Ericoids	0.11	0.117	0.219	0.071	0.466	0.118	0.19	0.144	0.189	

Finally, each family cluster overlaps with at least two other families from its own and other suprafamilial clades (PERMANOVA: *F* = 18.85, *r*^2^ = 0.52, *p* < 10^−4^, electronic supplementary material, table S1 and figure S3). For instance, Ericaceae significantly differ from only 11 of the 21 other families and distinctly overlap with, e.g. Cyrillaceae and Marcgraviaceae (electronic supplementary material, table S1).

### (b) Variation of disparity

The disparity of ericalean families ranged from 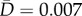 and *R* = 0.007 in Mitrastemonaceae to 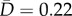 and *R* = 0.27 in Lecythidaceae.

Four ericalean families together contribute 50% of the Ericales disparity: Lecythidaceae (*PDiv* = 16.1%), Sapotaceae (*PDiv* = 14.3%), Primulaceae (*PDiv* = 14%) and Ericaceae (*PDiv* = 9.8%, electronic supplementary material, figure S4).

### (c) Correlations between disparity and clade size/clade age

**Figure 2.**
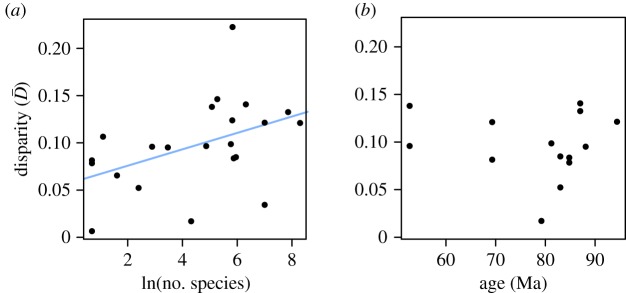
Relation between disparity and (*a*) species richness (log transformed) and (*b*) stem age in ericalean families. Blue line: linear regression between disparity and log transformed species number (Spearman's *rho* = 0.14, *p* = 0.63). (Online version in colour.)

We found a positive, but nonlinear, correlation between disparity (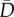) and the species richness of families (Spearman's *rho* = 0.49, *p* = 0.02; *R*: *rho* = 0.64, *p* = 0.001; figure 2*a*). We found no significant correlation between disparity (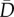) and the age of families (Spearman's *rho* = 0.14, *p* = 0.63; *R*: *rho* = −0.12, *p* = 0.68; figure 2*b*). With few exceptions (electronic supplementary material, figure S4), partial disparity significantly increased with species number (Spearman's *rho* = 0.84, *p* < 1.10^−5^).

### (d) Incorporation of floral fossils

The nine fossils of Ericales included in our dataset group together near the centre of the morphospace (figure 1*e*). Their distribution in the space overlaps with the balsaminoids, the ericoids, the Pentaphylacaceae-Sladeniaceae clade and Mitrastemonaceae (electronic supplementary material, table S2) and they contribute 1.8% to the total disparity of Ericales. Note that the percentage of missing data in the fossil dataset was 10.51% (electronic supplementary material, table S2).

### (e) Comparison of the disparity of floral functional modules

The most variable characters (i.e. characters with a mean pairwise difference between all taxa *Dchar* ≥ 0.5) stem from the androecium (anther orientation, filament fusion to corolla and anther attachment) whereas the least variable characters (i.e. characters with a mean pairwise difference between all taxa *Dchar* ≤ 0.05) describe the perianth (number of petal whorls, petal phyllotaxis, sepal phyllotaxis, and perianth differentiation; electronic supplementary material, figure S5).

**Figure 3.**
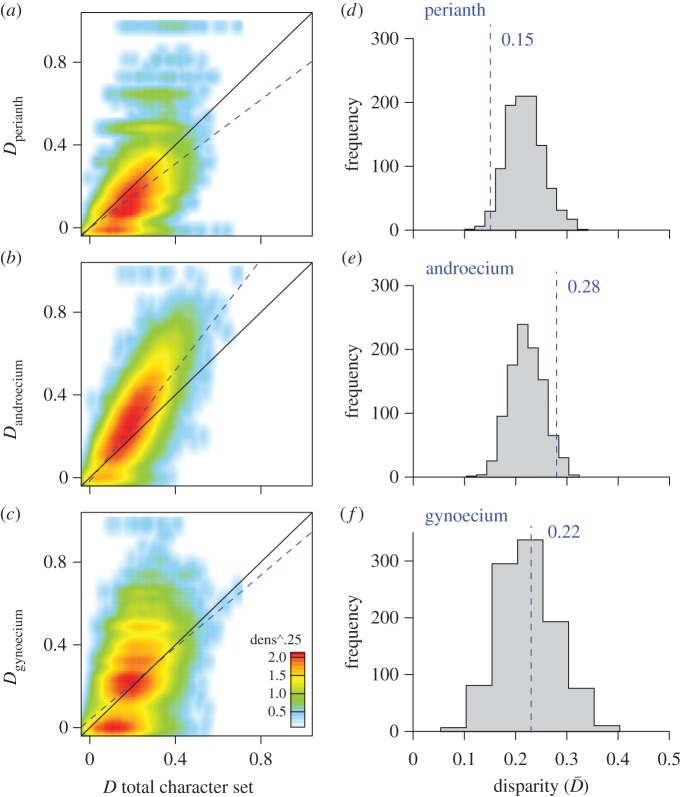
Differential variation in the three functional modules of Ericales flowers. (*a*–*c*) Pairwise dissimilarities (*D*) for (*a*) the perianth, (*b*), the androecium and (*c*) the gynoecium, plotted against pairwise dissimilarities for the total character set. Black plain lines: *y* = *x*. Blue dashed lines: linear regressions between the plotted variables. The heat colour gradient indicates the density of dots in the graph. (*d*–*f*) Disparity (in blue) of (*d*) the perianth, (*e*) the androecium and (*f*) the gynoecium, plotted with the distributions of 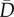 calculated for the total character set. (Online version in colour.)

Disparity for each of the three modules significantly increases with disparity of the whole character set (figure 3*a*–*c*): Mantel test for the perianth (71 631 taxa pairs): *p* < 0.001; for the androecium (72 390 taxa pairs): *p* < 0.001; for the gynoecium (70 876 taxa pairs): *p* < 0.001.

The perianth varies significantly less than the rest of the flower (mean pairwise distances between all taxa for the perianth organs only: 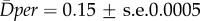; bootstrap analysis: *p* = 0.040; figure 3*a*,*d*), the androecium varies marginally more than the rest of the flower (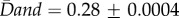; *p* = 0.073; figure 3*b*,*e*), and the gynoecium shows neither less nor more variation than the rest of the flower (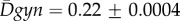; *p* = 0.444; figure 3*c*,*f*).

## 4. Discussion

### (a) The floral morphospace of Ericales

The floral morphospace of Ericales is organized in a continuous cloud, where most of the suprafamilial clades occupy distinct neighbouring regions arranged in a mosaic pattern. In other words, each of these lineages evolved towards a distinct combination of floral morphological traits. Our analysis also reveals two contrasting patterns of trait variation. On the one hand, ericalean species from across the order share recurrent combinations of character states: for instance, 75.1% of the 381 sampled species share structurally bisexual flowers with a differentiated perianth, a single whorl of sepals, and a single whorl of five petals (electronic supplementary material, table S2). Many of these common traits are likely plesiomorphic and evolutionarily constrained within the order. On the other hand, some floral traits, such as petal union and stamen and integument numbers, are highly variable in Ericales (see electronic supplementary material, table S2), although they have traditionally been considered stable within major angiosperm clades [35]. These two conflicting patterns are the main reasons for the pre-molecular phylogenetic placement of Ericales' taxa in more than 10 different angiosperm orders [44,49].

Within each suprafamilial clade, our analysis showed that there are two main patterns of space occupation by families. The balsaminoid, styracoid, sarracenioid and ericoid clades are all morphologically homogeneous, with the families overlapping (electronic supplementary material, table S1). For instance, the sarracenioids (Sarraceniaceae, Roridulaceae, and Actinidiaceae) are all characterized by, e.g. free petals to which stamens are not (or only basally) attached. The recovery of sarracenioids based on morphology is consistent with their phylogenetic relationships and illustrates a case where floral traits typically have a higher diagnostic value than vegetative traits [66]. In contrast with the pattern described just above, families of the polemonioid, primuloid and Pentaphylacaceae-Sladeniaceae clades occupy distinct regions of the morphospace (electronic supplementary material, table S2). For instance, in the polemonioids, flowers of Polemoniaceae and Fouquieriaceae significantly differ (see also [45]): Fouquieriaceae flowers have a free calyx and more than five stamens arranged in two whorls and free from the corolla, whereas Polemoniaceae flowers generally have a united calyx and a single whorl of five stamens that are more or less fused with the corolla. Consequently, Polemoniaceae and Fouquieriaceae were placed in various different orders before being recovered in molecular phylogenies [35,67]. A discussion about Ericales families whose placement in the phylogeny is not resolved is given in the electronic supplementary material.

### (b) Disparity

In Ericales, the most variable family, Lecythidaceae, is a pantropical family of trees (340 species). The least variable family, Mitrastemonaceae, is a root-parasitic family composed of only one Asian and one Central American species (it is thus the smallest family of Ericales). The positive correlation between disparity and the species richness of families can be explained by the fact that family size is highly variable in Ericales, ranging from two species in Mitrastemonaceae to 4010 in Ericaceae. Although disparity measured as mean pairwise distances is generally robust against differences in group sizes [60], a group containing a thousand times more species than another is very likely to be more diverse. Additionally, reproductive isolation is often due to differences in floral traits [68,69], hence a correlation between floral disparity and species number is, in many cases, to be expected.

In a study on the disparity of Neotropical pollen morphotypes, Mander [70] found a similar positive correlation between disparity and family size, with some exceptions (e.g. Poaceae and Papilionideae). Such exceptions to the overall patterns are also revealed in our study and may be illustrated by comparing, for instance, Sapotaceae and Lecythidaceae. Although Sapotaceae are more than three times as speciose as Lecythidaceae, their flowers are only half as morphologically diverse (figure 1). These two families are both constituted mainly of tropical trees, and have similar stem ages [33,42]. Other factors might thus explain their contrasted disparity, such as different diversification events, polyploidization events, species distribution or ecology. For instance, the higher diversity of Lecythidaceae may be linked to specialized floral adaptations towards different functional groups of pollinators. Lecythidaceae have evolved highly elaborate and specialized pollination mechanisms involving bees, beetles and bats [71–73], whereas Sapotaceae appear to be characterized by more generalist insect or sometimes bat pollination systems [42,74].

Only four ericalean families together (Lecythidaceae, Sapotaceae, Primulaceae and Ericaceae) contribute 50% of the Ericales disparity. Some families, like Ericaceae and Pentaphylacaceae, display high partial disparity because they are widely spread in the space; i.e. are themselves highly variable. Alternatively, some families, like Sapotaceae and Balsaminaceae, display high partial disparity because they are distributed in the periphery of the space, i.e. they increase the overall disparity by adding new traits or trait combinations. Such traits are, e.g. two whorls of sepals in Sapotaceae, and distally united filaments, nectar spur and zygomorphic flowers in Balsaminaceae (see electronic supplementary material, table S2). Zygomorphy and the presence of nectar spurs might explain the peculiar pattern found in Balsaminaceae: the family displays very low morphological disparity in the investigated organizational floral traits (figure 1*b*), in spite of the fact that it is extremely speciose. Balsaminaceae is composed of two genera: *Impatiens* (1100 spp.) and *Hydrocera* (1 sp.). The high taxonomic diversity in *Impatiens* may result from rapid radiation during the Pliocene and Pleistocene, triggered by climatic fluctuations resulting in refuge areas [75]. This recent diversification might explain the low structural disparity in Balsaminaceae (although perianth shape and colour, not coded here, are highly variable [76]). Zygomorphy and nectar spurs are often considered to be key innovations associated with high diversification rates: they are often linked to speciation through increased specialization in pollination, e.g. through precise pollen placement on pollinator bodies [77,78], and spur length filtering for the most efficient pollinators [79].

The lack of correlation between disparity and the age of families is not surprising, as disparity does not necessarily steadily increase over time [80]. The nine fossils we included showed low levels of disparity and contributed little to the total disparity of Ericales. These fossils are not considered to be closely related to each other [8,40], and have been tentatively assigned to a number of different families, albeit all belonging to a group consisting of ericoids, sarracenioids, styracoids and Pentaphylacaceae-Sladeniaceae (the assigned positions of the fossils, according to original papers, are highlighted in figure 1*a*). They are close in age (72–94 Ma) to the Ericales' initial diversification (crown age: *ca* 104 Ma [33]) and the features they share (e.g. bisexual flowers, pentamerous and actinomorphic whorls of sepals and petals, free stamens, and superior ovaries) could thus represent plesiomorphies for the order. Compared with other types of fossils (e.g. permineralized fossils or compression/impression fossils), charcoalified fossils often show excellent preservation of morphological and anatomical features [8]. The availability of such fossils thus offers opportunities for future work on the extinct disparity of flowers, also at the level of the angiosperms as a whole.

### (c) Linking function and disparity in the flower

Our results indicate that, in Ericales, morphological variation differs considerably among the flowers' three functional modules, probably because of the different selective regimes they are submitted to. The perianth varied significantly less than the rest of the flower, as expected. Contrastingly, the androecium varied marginally more than the rest of the flower, and the gynoecium showed neither less nor more variation than the rest of the flower, although we expected it to show more variation, due to its complex organization.

In our dataset, most species are characterized by actinomorphic, whorled, and pentamerous flowers (like most core eudicots [5,10]). In general, the perianth is structurally less complex than the reproductive floral organs [27] and the characters we could code for were mostly related to organ number and arrangement (Bauplan; [81]). In the perianth, these characteristics are likely spatially and functionally constrained during development and anthesis and mostly stable at higher taxonomic ranks, with one explanation being that the number of perianth organs and their arrangement depends on meristem size during early development [10]. Stamen number, however, appears to be much less constrained, ranging from two to several hundred in our dataset (see electronic supplementary material, table S2). Large stamen numbers can easily be accommodated even on a relatively small floral base as the filament bases are generally small [7]. Polystemony (i.e. flowers with more stamens than perianth organs) has apparently evolved along several separate lineages in Ericales [35]. In addition to the diversity in stamen numbers, ontogenetic patterns of androecium development and anthetic stamen arrangement are also particularly diverse in Ericales, including complex ring primordia with multiple stamen whorls and stamens arranged in fascicles [82]. With the exception of early diverging angiosperms, there are probably only few other groups of angiosperms with such labile and diverse patterns of stamen numbers and arrangement as the Ericales. It seems likely that this lability in the androecium has played a major role during the evolutionary history and diversification of the Ericales and has allowed the group to explore new evolutionary paths in connection with different functional groups of pollinators.

Finally, the gynoecium, with its multiple functions and complex morphogenesis, is often considered the most complex module of the flower [7,32]. All Ericales in our dataset were syncarpous. Syncarpy is relatively stable in angiosperms [81] and is believed to have many advantages [83]. For instance, it allows for the presence of a centralized canal for pollen germination that allows a single pollen load on a stigma to potentially reach all the ovules of the same flower [83]. It has been proposed that syncarpy allows for higher levels of synorganization both within the gynoecium and also between the gynoecium and other floral organs [32], like in Balsaminaceae and Tetrameristaceae, where the syncarpous gynoecium is highly synorganized with the androecium [46]. Once syncarpy has evolved, it is therefore likely to remain stable, and it is a factor decreasing disparity in the gynoecium. However, other gynoecial traits such as ovary position, type of placentation, the number of ovules per carpel and the number of integuments are remarkably variable across Ericales (electronic supplementary material, table S2). These contrasting trends of variation might explain the lack of signal for more or less variation of the gynoecium.

Overall, variability occurs less at the level of floral organization (e.g. organ number, organ arrangement), than at the level of floral construction (architecture, mechanical properties) and mode (traits like organ shape and colour). Floral mode often directly concerns interactions with pollinators and is generally highly variable, even at low taxonomic ranks [81,84]. Such traits could not be included in our analysis, as most of them would not have been applicable throughout the order. Our dataset is more representative of floral organization and construction, with some exceptions: the most variable characters in the perianth are the union of sepals and of petals (electronic supplementary material, figure S5), typically linked to pollination, allowing for the formation of corolla tubes and a canalized access to floral rewards [85], channelling pollinator movements so that they touch stamens and stigmas [77].

## Supplementary Material

Supplementary information

## Supplementary Material

Supplementary figures

## Supplementary Material

Table S2

## Data accessibility

The dataset supporting this article has been uploaded as part of the supplementary material (electronic supplementary material, table S2).
