# Supplementary material for: How (much) do flowers vary? Unbalanced disparity among flower functional modules and a mosaic pattern of morphospace occupation in the order Ericales
Source: Proc Biol Sci. 2017 Apr 5;284(1852):20170066. doi: 10.1098/rspb.2017.0066 (PMC5394665; doi:10.1098/rspb.2017.0066)
Supplement: Supplementary information [file rspb20170066supp1.pdf]

Contents

1. Abbreviations and terminology 1
2. Table S1 2
3. Characters and character states 3
4. Supplementary discussion about Ericales families whose placement in the phylogeny is not resolved 9
5. Supplementary figure legends 10
6. Supplementary table legends 10
7. References 11

1. Abbreviations and terminology

N	total number of characters
d	difference between two taxa for one character
d_{ABi}	d for taxa A and B, and character i
D	mean character difference = mean(d) for a pair of taxa
D_{AB}	D for taxa A and B
PCoA	principal coordinate analysis
$\overline{D}$	disparity = mean pairwise dissimilarity = mean(D) for a group of taxa
$\overline{D}per$	$\overline{D}$ calculated from the character set describing the perianth
$\overline{D}and$	$\overline{D}$ calculated from the character set describing the androecium
$\overline{D}gyn$	$\overline{D}$ calculated from the character set describing the gynoecium
p	pseudo p-values or p-values
PDiv	partial disparity
R	range (maximum pairwise dissimilarity) = max(D) for a group of taxa
Dchar	mean taxa difference (mean d) for a single character

2. Table S1

Table S1. Post hoc pairwise comparisons (PERMANOVA) based on floral traits among Ericales families.

	Actinidiaceae	Balsaminaceae	Clethraceae	Cyrillaceae	Diapensiaceae	Ebenaceae	Ericaceae	Fouquieriaceae	Lecythidaceae	Marcgraviaceae	Mitrastemonaceae	Pentaphylacaceae	Polemoniaceae	Primulaceae	Roridulaceae	Sapotaceae	Sarraceniaceae	Sladeniaceae	Styracaceae	Symplocaceae	Tetrameristaceae	Theaceae
Actinidiaceae	NA	28.15	24.32	7.35	26.60	19.25	15.70	20.58	17.86	14.52	9.03	10.19	28.03	21.72	3.79	45.22	5.06	6.77	15.54	16.65	6.94	4.59
Balsaminaceae	0.597	NA	164.14	26.74	79.10	36.40	41.92	55.32	10.14	39.35	11.92	36.98	47.23	40.58	40.27	68.39	45.77	41.64	43.88	56.62	49.21	23.39
Clethraceae	0.5614	0.9012	NA	3.99	29.80	55.48	5.12	11.92	62.38	4.64	71.53	5.79	32.71	20.53	3.28	63.78	18.21	13.48	25.01	27.21	11.62	19.74
Cyrillaceae	ns	ns	ns	NA	3.31	9.72	1.07	1.28	18.89	0.57	5.91	1.04	5.14	3.24	0.50	12.96	5.37	1.23	5.35	4.81	0.76	5.68
Diapensiaceae	0.5833	0.8146	0.6235	ns	NA	19.29	4.37	4.86	55.05	4.65	19.71	6.49	6.70	4.84	4.83	17.06	23.67	3.10	13.39	9.56	7.59	23.43
Ebenaceae	0.4905	0.657	0.7449	ns	0.5038	NA	18.29	19.94	19.83	18.37	8.66	14.63	10.98	10.73	8.99	14.57	25.57	6.15	11.73	11.56	12.02	16.81
Ericaceae	0.197	0.3995	ns	ns	ns	0.2222	NA	3.41	76.17	2.31	9.04	6.70	13.92	19.53	0.94	61.63	10.01	1.56	14.05	8.27	2.04	19.75
Fouquieriaceae	0.52	0.7545	0.3984	ns	ns	0.512	ns	NA	49.57	1.69	13.35	4.58	10.65	6.95	2.11	33.25	14.32	2.22	11.01	8.66	2.69	15.57
Lecythidaceae	0.3316	0.2246	0.6406	0.4029	0.6113	0.3551	0.4877	0.5862	NA	47.26	3.41	47.71	56.13	70.60	10.24	105.71	24.37	13.12	32.38	29.86	14.67	16.48
Marcgraviaceae	ns	0.6862	ns	ns	ns	0.4916	ns	ns	0.5745	NA	8.68	3.06	12.02	10.13	0.98	38.39	8.97	1.75	12.30	8.42	1.43	13.59
Mitrastemonaceae	ns	0.5439	0.8773	ns	ns	0.4404	ns	0.5717	ns	ns	NA	8.46	10.03	7.97	40.29	13.14	14.27	16.69	10.41	14.96	36.79	6.19
Pentaphylacaceae	ns	0.5691	ns	ns	0.1882	0.3353	0.0841	ns	0.5146	ns	0.2972	NA	17.06	14.29	0.65	43.43	6.41	0.88	10.10	5.76	0.76	10.61
Polemoniaceae	0.5094	0.6449	0.5572	ns	ns	ns	0.164	0.2907	0.5662	0.3162	0.3578	0.3215	NA	4.85	4.95	19.58	24.58	3.56	14.99	11.92	7.36	28.18
Primulaceae	0.2448	0.3808	0.2373	ns	ns	0.138	0.1496	0.0952	0.4596	0.133	ns	0.1583	ns	NA	2.90	33.51	17.31	1.51	14.00	9.13	3.90	23.86
Roridulaceae	ns	ns	ns	ns	0.3258	ns	ns	ns	0.2749	ns	ns	ns	ns	ns	NA	9.65	3.04	2.39	3.10	3.84	2.59	2.89
Sapotaceae	0.3858	0.4906	0.4732	0.1684	0.1937	0.1683	0.3469	0.319	0.5457	0.3509	0.1725	0.349	0.1987	0.2197	ns	NA	42.83	7.07	41.13	23.16	13.37	54.36
Sarraceniaceae	ns	0.7177	0.5029	ns	0.568	0.5737	0.1371	0.4431	0.4105	ns	0.5879	ns	0.486	0.2078	ns	0.3763	NA	6.62	14.36	16.41	4.86	4.01
Sladeniaceae	ns	0.7911	0.5507	ns	ns	0.339	ns	ns	ns	ns	ns	ns	ns	ns	ns	ns	ns	NA	3.69	3.56	3.16	4.53
Styracaceae	0.357	0.6191	0.4808	ns	0.3316	0.2952	0.1633	0.2896	0.4239	0.313	0.354	0.2144	0.2998	0.1573	ns	0.3395	0.3472	ns	NA	2.67	5.63	11.28
Symplocaceae	0.4543	0.7487	0.5888	ns	ns	0.3664	ns	ns	0.4534	ns	ns	ns	0.3062	0.1199	0.2588	0.2434	0.4634	ns	ns	NA	6.43	12.87
Tetrameristaceae	ns	ns	0.5137	ns	0.4083	0.5003	ns	ns	0.3438	ns	ns	ns	ns	ns	ns	0.1728	ns	ns	ns	ns	NA	3.71
Theaceae	ns	0.5042	0.4618	ns	0.5046	0.4119	0.225	0.4037	0.2918	0.3714	0.2922	0.2433	0.4761	0.2515	ns	0.417	ns	ns	0.2607	0.3491	ns	NA

F (upper diagonal) and r^2 (lower diagonal) values are given for significantly different comparisons. ns = families that are not significantly different. Overall test: PERMANOVA: $F = 18.85$, $r^2 = 0.52$, $p < 10^{-4}$.

3. Characters and character states

Most of the terminology used here follows Endress (1996).

GENERAL TRAITS:

1. Functional sex: Characterizes individual plants and not individual flowers. Here we treat all plant individuals with functionally unisexual flowers as unisexual. These may include individuals with structurally bisexual flowers (e.g. male flowers with pistillodes and female flowers with staminodes as present in Actinidiaceae). (0) individuals with functionally bisexual flowers only; (1) individuals with functionally unisexual flowers only; (2) individuals with functionally unisexual and bisexual flowers.

2. Structural sex: Characterizes individual plants and not flowers. Here we treat all plant individuals with male and female organs as bisexual, irrespective of whether the organs are functional or non-functional (e.g. the functionally unisexual flowers of many Actinidiaceae are scored bisexual as functionally male or female flowers always bear sterile organs of the opposite sex). (0) individuals with only bisexual flowers; (1) individuals with only unisexual flowers (no remnants of the opposite sex); (2) both types of flowers can be found (e.g. *Ecclinusa ulei* in Sapotaceae, with functionally unisexual flowers, the male flowers structurally bisexual as presenting vestigial ovules, Pennington 1990).

3. Nectaries and elaiophores: Here, we scored for the presence of nectar and oil (as a pollinator reward) producing structures. In Ericales, such structures may correspond to perianth spurs, nectary discs, nectar producing trichomes or staminodes. Detailed information on the presence/position/structure of nectaries and elaiophores is nevertheless very scarce in literature; therefore we kept this character binary: (0) absent; (1) present.

4. Flower length: In cases where length was described as a range, we scored length as the mean of this range. (0) small (≤1cm); (1) medium sized (>1cm and ≤3cm); (2) large: (>3cm).

5. Flower diameter: In cases where the diameter was described as a range, we scored diameter as the mean of this range. (0) small (≤1cm); (1) medium sized (>1cm and ≤3cm); (2) large: (>3cm).

PERIANTH:

6. Perianth differentiation: Typically, outer perianth parts are sepaloid and protect the other floral organs during floral development, while inner organs are often petaloid and play a role in pollinator attraction. However, it is also possible that all parts are

either sepaloid or petaloid but remain differentiated in shape, size, and/or texture. In case of spiral perianth organ arrangement, differentiation may be continuous (i.e. gradual), whereby two successively initiated organs are very similar or only slightly different, while the outermost and innermost organs at both ends of the spiral are very different from each other (e.g. Theaceae). Within-whorl differentiation, whereby organs of the same whorl take different forms, occurs for instance in zygomorphic flowers (e.g. Balsaminaceae) but is not taken into account here. (0) No differentiation or weak differentiation (a single case, *Calluna vulgaris* in Ericaceae, has similar sepals and petals slightly differing in size and shape, Small 1914); (2) continuous differentiation, outer sepaloid, inner petaloid; (3) marked differentiation: outer sepaloid and inner petaloid or marked differentiation among petaloid tepals or marked differentiation among sepaloid tepals or marked differentiation with calyx the same color as the petals.

7. Number of sepals: Numerical. Not scored in the case of continuous differentiation between sepals and petals.

8. Number of petals: Numerical. Not scored in the case of continuous differentiation between sepals and petals.

9. Sepal phyllotaxis: In most Ericales, sepals are organized as one or more whorls. In 2 species (*Laplacea alpestris* (Melchior 1925) and *Stewartia pseudocamellia* (Erbar 1986), in Theaceae), the perianth is organized along a continuous spiral. (0) whorled; (1) spiral.

10. Petal phyllotaxis: As for character 35 (Sepal phyllotaxis). A few species (e.g. *Laplacea alpestris* (Melchior 1925) and *Stewartia pseudocamellia* (Erbar 1986), in Theaceae) have a spiral sepal phylllotaxis. (0) whorled; (1) spiral.

11. Number of sepal whorls: Numerical. Most Ericales have a single whorl of sepals, with the exception of some Sapotaceae and *Marcgravia umbellata* in Marcgraviaceae (Caris 2013), which have two whorls of sepals.

12. Number of petals whorls: Numerical. Most Ericales have a single whorl of petals, with the exception of *Allotropa virgata* in Ericaceae (Lichthardt and Mancuso 1991) and *Foetidia obliqua* in Lecythidaceae (Tsou 1994), that have no petals, and *Archboldiodendron calosericeum* in Pentaphylacaceae (Kobuski 1940) that has two whorls of petals. Flowers of *Gustavia macarenensis* (Sapotaceae) have 6-8 petals arranged in one whorl when their number is 6 or 7; these petals arise in two whorls when their number is 8 (Tsou and Mori 2007). We thus scored the species as polymorphic for this character.

13. Sepal aestivation: Aestivation refers to the mutual position of perianth organs. As aestivation patterns might not be visible at anthesis, this character was preferably coded from information recorded for pre-anthetic flowers. Aestivation may be apert (neighbouring organs do not touch), imbricate (neighbouring organs overlap) or valvate (margins of neighbouring organs touch but do not overlap). (0) apert; (1) imbricate; (2) valvate.

14. Petal aestivation: As for character 13 (sepal aestivation). (0) apert; (1) imbricate; (2) valvate.

15. Perianth symmetry: There are many ways in which flowers can be monosymmetric (i.e. zygomorphic, with a single plane of bilateral symmetry). Here we record perianth symmetry, regardless of androecium or gynoecium symmetry. In addition, disymmetry (two orthogonal planes of bilateral symmetry (e.g. *Marcgravia umbellata* in Marcgraviaceae; Caris 2013) and asymmetry (a single case: *Pelletiera wildpretii* in Primulaceae; Valdés 1980) are treated here as separate character states. (0) polysymmetric; (1) monosymmetric; (2) disymmetric; (3) asymmetric.

16. Sepal union: Here, we scored sepal union as a binary character with a threshold at 10%. The rationale for the 10 % threshold is that organs of the same whorl or even of two successive whorls often appear to have a short common base (which may be interpreted as part of the receptacle) and accordingly are often described as 'basally connate' or 'basally adhering' without being clearly united. (0) free or basally united (0-10% union); (1) more than 10% union.

17. Petal union: As for sepal union (character 16). (0) free or basally united (0-10% union); (1) more than 10% union.

18. Distal filament union: In some cases, filaments may be free from each other at their base but may be postgenitally united distally (e.g. *Impatiens* in Basalminaceae; von Balthazar and Schönenberger 2013). (0) absent; (1) present.

ANDROECIUM:

19. Number of stamens: *Numerical.* Number of fertile stamens (not staminodes). Scored for the male flowers in the case of species with unisexual flowers. Scored as missing data when described as 'numerous' in the literature (20 cases). In Mistrastemonaceae, stamens are totally fused. We estimated the number of stamens for *Mitrastemon yamamotoi* as the number of vascular bundles in the androecium (Flora of China 2014).

20. Androecium organisation: In Ericales, androecium organization is highly diverse, especially in connection with polystemony. Here, we distinguish the following types. (0) whorled; (1) whorled with stamens in pairs; (2) spiral; (3) fascicles (whorled with groups of more than two stamens each); (4) ring primordium (individual stamens primordia emerge on a complex ring primordium).

21. Number of androecium whorls: We considered fertile stamens as well as staminodes (excluding sterile stamens from unisexual flowers) for this character. Furthermore, in cases of stamen fascicles (e.g. *Gordonia lasianthus* in Theaceae; Sargent 1891), it is the whorls of fascicles that we record here. (0) one; (1) two; (2) more than two.

22. Androecium symmetry: Here, we recorded androecium symmetry, regardless of perianth or gynoecium symmetry. Different floral organ categories may exhibit different symmetry patterns. For instance in many Lecythidaceae, androecium may

be distinctly monosymmetric while the perianth and the gynoecium are polysymmetric. (0) polysymmetric; (1) monosymmetric.

23. Filament union: Only fertile stamens were taken into account. (0) free or basally united (0-10% union); (1) more than 10% union.

24. Anther union: (0) free; (1) united to some extent.

25. Anther orientation: At anthesis, anther stomia (the two pollen sacs of each theca open through a common stomium) may face the floral center (i.e. they are introrse) or they face the floral periphery (i.e. they are extrorse), which also designates their direction of dehiscence. A third possibility are anthers which open their thecae toward the side (i.e. toward their neighbouring anthers; latrorse). Last, thecae may also open at the tip of the anther and thus they dehisce upward in the flower (apical). (0) introrse; (1) latrorse; (2) extrorse; (3) apical.

26. Anther attachment: Anther attachment refers to the insertion of the filament on the anther connective (i.e. the tissue connecting the two thecae of an anther). Anthers may be basifixed, with the filament attached to the base of the connective; dorsifixed, with the filament attached to the dorsal side of the anther, or ventrifixed, with the filament attached to the ventral side of the anther. (0) basifixed; (1) dorsifixed; (2) ventrifixed.

27. Anther dehiscence: Anther dehiscence refers to the type of opening of the anther when releasing its pollen. The most common mode of dehiscence is by longitudinal slits that extend along the entire length of each theca. In Ericales, dehiscence may also be restricted to shorts slits or pores either located at the morphological tip or at the morphological base of an anther. The two thecae of an anther may also be confluent distally and have a common, longitudinal stomium. In rare cases, anthers may also dehisce via short transversal slits (here a single case: *Pyxidanthera barbulata* in Diapensiaceae; Palser 1963). (0) longitudinal slit; (1) apically poricidal (pore or short slits); (2) basally poricidal (pores or short slits); (3) common stomium of confluent thecae; (4) transverse (horizontal) slit.

28. Filament insertion to corolla: In sympetalous flowers, the stamen filaments are often inserted on the corolla tube. Here, we scored the percentage of total corolla length that is fused to the filament (i.e. the height of the petal at which the stamens are inserted). When there were several whorls of stamens, filament insertion to corolla was calculated for each whorl and, if needed, coded as polymorphic. We scored this character for fertile stamens only. (0) no fusion (0-0.05%); (1) inserted in the lower half corolla; (2) inserted in the upper half of corolla.

29. Filament fusion to corolla: In sympetalous flowers, the stamen filaments are often inserted on the corolla tube. Here, we scored the percentage of total filament length that is fused to the corolla. When there were several whorls of stamens, the percentage of fusion to corolla was calculated for each whorl and, if needed, coded as polymorphic. We scored this character for fertile stamens only. (0) no fusion or only basally attached (less than 10%); (1) more than 10% fusion.

30. Staminodes: Here, we scored different types of staminodes (sterile stamens). We did not consider sterile stamens in functionally female flowers. (0) fused to form a corolla like structure; (1) stamen like (i.e. similar in shape as fertile stamens); (2) hood-like structure; (3) scale-like; (4) petaloid (no filament-anther differentiation, but like a petal with marginal microsporangia); (5) triangular, wooly or hairy, applied to the style at anthesis (e.g. *Mimusops elengi* in Sapotaceae; Aubréville 1963); (6) absent; (8) petal-like (petaloid without microsporangia).

GYNOECIUM:

31. Ovary position: The ovary is the part of the gynoecium where the ovules are produced. The ovary may be located on the receptacle and thus be positioned above the insertion level of the remaining floral organs (i.e. the ovary is superior and the flower is hypogynous). Alternatively the ovary may be embedded into the receptacle and therefore be located below the insertion level of the remaining floral organs (i.e. the ovary is inferior and the flower is epigynous). It is also possible that the ovary is inferior to a certain degree only, such as half-inferior, if the receptacle is surrounding the ovary to its mid-level (e.g. *Maesa japonica* in Primulaceae; Anderberg and Stahl 1995). Here we treat ovary position as a binary character, distinguishing only superior and inferior ovaries, and conservatively included all intermediate inferior states (incl. half-inferior) in the character state inferior. (0) inferior; (1) superior.

32. Number of carpels: *Numerical.* Consistent with our treatment of sexual dimorphism, the number of pistillodes in male flowers is ignored for this character. In multicarpellate, unilocular gynoecia with complete carpel fusion up to the stigma (e.g. *Primula* in Primulaceae; Douglas 1936), it may be difficult to unequivocally assess the number of carpels. In such cases, we have scored the number of carpels only if it is well established based on anatomical or developmental investigations. Similarly, in gynoecia where one or more carpels are reduced (e. g., in the gynoecium of *Pelliciera* in Tetrameristaceae; von Balthazar and Schönenberger 2013), the total number of structural carpels was only scored when unequivocally determined in the literature. As ovaries are nearly always united in Ericales, descriptions often only refer to the number of 'locules' (e.g. in Kubitzki et al. 2004). We assumed the number of locules to be the same as the number of carpels if there was no mention of 'secondary', 'additional' or 'false' septa.

33. Gynoecium symmetry: Here, we recorded gynoecium symmetry, regardless of perianth or androecium symmetry. Different floral organ categories may exhibit different symmetry patterns. For instance in many Lecythidaceae, androecium may be distinctly monosymmetric while the perianth and the gynoecium are polysymmetric. (0) polysymmetric; (1) monosymmetric; (2) disymmetric.

34. Style union: Here, we considered the length of the styles as the distance between the top of the ovary and the stigmatic surface. (0) free or basally united (0-10% union); (1) more than 10% union.

35. Placentation: In syncarpous ovaries, placentation is often axile in the synascidiate (and basal symplicate) region of the ovary (i.e. the carpel margins are united in the centre of the ovary). However, in the symplicate region of the ovary, carpel margins often do not meet and placentation therefore appears intruding-parietal. These cases were consistently scored as axile. Only truly parietal placentation (no axile region) was scored as such. Both, axile and parietal placentation are clearly distinguished from free-central placentation as it is present in Primulaceae, where the ovules are attached to a structure originating from the base of the syncarpous, uni-locular ovary. (0) axile; (1) free-central; (2) parietal.

36. Number of ovules per carpel: Number of ovules per functional carpel. As the number of ovules is not necessarily the same as the number of seeds, reports of seed numbers were not considered for this character. (0) one; (1) two; (2) three to ten; (3) more than ten.

37. Number of ovule integuments: In *Impatiens parviflora* and *I. auricoma* (Basalminaceae; von Balthazar and Schönenberger 2013), integuments are intermediate: there are two integuments during early development, but then the base of both integuments grows, so that it is difficult to discern the two integuments in mature ovules (see McAbee et al. 2005); we scored theses species as bitegmic. (0) one (unitegmic); (1) two (bitegmic).

4. Supplementary discussion about Ericales families whose placement in the phylogeny is not resolved

The exact phylogenetic positions of the three families Theaceae, Lecythidaceae, and Mitrastemonaceae, as well as the position of the clade with Pentaphylacaceae and Sladeniaceae, are still equivocal. In our analysis, the morphospace distribution of Theaceae overlaps with that of the sarracenioids, mainly due to the similarities in floral structure with Actinidiaceae (Table S2). These two families share a high number of stamens, five free petals and sepals, and medium to large flowers (Löfstrand & Schönenberger, 2015). The phylogenetic proximity of Theaceae and Actinidiaceae has earlier been proposed based on morphology (Cronquist, 1981). In molecular analyses, Theaceae forms a polytomy with the styracoids, sarracenioids, and ericoids (Schönenberger *et al.*, 2005). The similarity of Theaceae and Actinidiaceae flowers may be explained by evolutionary convergence due to similar selective regimes, or by their close, albeit not immediate, phylogenetic relationship. Mitrastemonaceae did not significantly differ from half of the other clades (Table 1); they have a peculiar floral morphology with a completely united and undifferentiated perianth and stamens united into a cap-like structure covering the gynoecium during the male phase of anthesis (Meijer & Veldkamp, 1993). This led to a high percentage of inapplicable data (treated as missing) for the two species of this family and explains this non-significant result. Lecythidaceae differ significantly from all other clades except Mitrastemonaceae (Table 1) and clearly holds an isolated and, simultaneously, extensive region of the space. It is the most diverse family in Ericales and has evolved unique combinations of floral traits (Fig. 1; Fig. S3; see main discussion).

5. Supplementary figure legends

Fig. S1. Schematic representation of an angiosperm flower (longitudinal section), showing the different organs of the perianth (sterile part), androecium (male part), and gynoecium (female part).

Fig. S2. Sample size per family. Black horizontal bars indicate the percentages of species (A) and genera (B) sampled per family relative to the total taxon numbers accepted in The Plant List (The Plant List 2013, http://www.theplantlist.org). For each family, sample size is given in black above each bar and the accepted taxon number is given in blue below each bar.

Fig. S3. 3-dimensional PCoA representation of the morphospace of Ericales. Each family and species can be displayed by selecting the corresponding level in the arborescence (levels can be expanded by clicking on the '+ Levels' box in the 'Toggle Model Tree' tool of the software Adobe Acrobat). Selecting only the level called 'Ericales' displays the total morphospace and axes.

Fig. S4. Partial disparity [i.e. additive contribution (in %) of each family to the total disparity] and contribution (in %) to taxonomic diversity for the 22 Ericalean families.

Fig. S5. Character variation (*Dchar*), calculated as the mean pairwise difference between taxa for each character. Colour dots give D. Bars indicate standard errors. In yellow: characters describing the perianth, cyan: androecium, magenta: gynoecium, black: general features. The corresponding boxplots (displaying the median d_{AB} for each taxa pair, per character) are displayed in grey.

6. Supplementary table legends

Table S1. Post hoc pairwise comparison based on floral traits (PERMANOVA) among the ericalean families. ns = clades that are not significantly different. * clades that are significantly different. Overall test: F = 18.85, r2 = 0.52, p < 0.0001.

Table S2. Dataset used for the study, and composition of the functional modules.

7. References

Anderberg AA, Stahl B (1995). Phylogenetic interrelationships in the order Primulales, with special emphasis on the family circumscriptions. *Canadian J Bot* 73:1699–1730.

Anderson MJ (2001). A new method for non-parametric multivariate analysis of variance. *Austral ecology* 26:32-46.

Aubréville A (1963). *Flore du Cambodge, du Laos et du Vietnam*. Fascicule 3: Sapotaceae. Museum National d'Histoire Naturelle, Paris. 105pp.

Caris P (2013) Bloemontogenetische patronen in de Ericales sensu lato. PhD thesis, KU Leuven.

Ciampaglio CN, Kemp M, McShea DW (2001) Detecting changes in morphospace occupation patterns in the fossil record: characterization and analysis of measures of disparity. *Paleobiology* 27:695-715.

Crepet WL, Nixon KC, Daghlian CP (2013) Fossil Ericales from the Upper Cretaceous of New Jersey. *Int J Plant Sci* 174(3):572-584.

Cronquist A. 1981. *An integrated system of classification of flowering plants.* New York, Columbia University Press. (ISBN:0231038801)

Douglas GE (1936) Studies in the vascular anatomy of the Primulaceae. *Am J Bot* 23:199-212.

Endress PK (1996) *Diversity and evolutionary biology of tropical flowers* (Cambridge University Press, Cambridge).

Erbar C (1986). Untersuchungen zur Entwicklung der spiraligen Blüte von Stewartia pseudocamellia (Theaceae). *Botanische Jahrbücher für Systematik*. 106:391-407.

Flora of China (2014) http://www.efloras.org

Foote M (1992a) Rarefaction analysis of morphological and taxonomic diversity. *Paleobiology* 18(01):1-16.

Foote M (1992b) Paleozoic record of morphologica diversity in blastozoan echinoderms. *Proc Nat Acad Sciences* 89(16):7325-7329.

Foote M (1993) Contributions of individual taxa to overall morphological disparity. *Paleobiology* 19(04):403-419.

Foote M (1999) Morphological diversity in the evolutionary radiation of Paleozoic and post-Paleozoic crinoids. *Paleobiology* 25(S2):1-115.

Friis EM (1985) *Actinocalyx* gen. nov., sympetalous angiosperm flowers from the Upper Cretaceous of southern Sweden. *Rev Palaeobot Palynol* 45(3-4):171-183.

Keller JA, Herendeen PS, Crane PR (1996) Fossil flowers of the Actinidiaceae from the Campanian (Late Cretaceous) of Georgia. *Am J Bot* 83:528-541.

Klingenberg CP (2009). Morphometric integration and modularity in configurations of landmarks: tools for evaluating a priori hypotheses. *Evolution & development* 11:405-421.

Kobuski CE (1940). Studies in Theaceae, V. The Theaceae of New Guinea. *J. Arnold Arbor.* 21(2): 140-142.

Kubitzki K (2004) *The families and genera of vascular plants. Volume 6. Flowering plants: Dicotyledons: Celastrales, Oxalidales, Rosales, Cornales, Ericales* (Springer, Berlin).

Lichthardt J, Mancuso M (1991). Report on the conservation status of *Allotropa virgata* (candystick) on the Nez Percé National Forest. I. Field survey and first-and second-year monitoring results. Conservation Data Center, Nongame and Endangered Wildlife.

Löfstrand SD, Schönenberger J. 2015. Comparative floral structure and systematics in the sarracenioid clade (Actinidiaceae, Roridulaceae and Sarraceniaceae) of Ericales. *Bot J Linean Soc* 178, 1-46. (doi:10.1111/boj.12266)

Magallón S, Gómez-Acevedo S, Sánchez-Reyes LL, Hernández-Hernández T (2015) A metacalibrated time-tree documents the early rise of flowering plant phylogenetic diversity. *New Phytol* 207:437-453.

Martínez-Millán M, Crepet WL, Nixon KC (2009) *Pentapetalum trifasciculandricus* gen. et sp. nov., a thealean fossil flower from the Raritan Formation, New Jersey, USA (Turonian, Late Cretaceous). *Am J Bot* 96(5):933-949.

McAbee JM, Kuzoff RK, GasserCS (2005). Mechanisms of derived unitegmy among Impatiens species. *The Plant Cell* 17:1674-1684.

Meijer W, Veldkamp JF (1993) A revision of *Mitrastema* (Rafflesiaceae). *Blumea* 38(1):221-229.

Melchior H (1925). *Theaceae*. In: Engler A, ed. Die Natürlichen Pflanzenfamilien, 2nd ed. 21. Leipzig: Engelmann, 109-154.

Nixon KC, Crepet WL (1993) Late Cretaceous fossil flowers of ericalean affinity. *Am J Bot* 80:616-623.

Pennington TD (1990). Flora Neotropica, Monograph 52. Sapotaceae. New York: New York Botanical Garden.

R Core Team (2013). R: A language and environment for statistical computing. R Foundation for Statistical Computing, Vienna, Austria. URL   http://www.R-project.org/.

Sargent CS (1891). The Silva of North America, vol. 1: t. 21 [C.E. Faxon].

Sauquet H. 2016. PROTEUS: A database for recording morphological data and creating NEXUS matrices. Version 1.26. http://eflower.myspecies.info/proteus.

Schönenberger J, Friis EM (2001) Fossil flowers of ericalean affinity from the Late Cretaceous of Southern Sweden. *Am J Bot* 88(3):467-480.

Schönenberger J, Anderberg AA, Kenneth JS (2005) Phylogenetics and patterns of floral evolution in the Ericales. *Int J Plant Sci* 166(2):265-288.

Schönenberger J, von Balthazar M, Takahashi M, Xiao X, Crane PR, Herendeen PS (2012) *Glandulocalyx upatoiensis*, a fossil flower of Ericales (Actinidiaceae/Clethraceae) from the Late Cretaceous (Santonian) of Georgia, USA. *Ann Bot* 109(5):921-936.

Small JK (1914). *Ericaeceae*. In: Small JK, ed. North American Flora 29/1. New York: The New York Botanical Gardens, 33-102.

Stevens PF (2001 onwards) Angiosperm Phylogeny Website. Version 12, July 2012. [WWW document] http://www.mobot.org/MOBOT/research/APweb/welcome.html [accessed 1 January 2014].

The Plant List (2013). Version 1.1. Published on the Internet; http://www.theplantlist.org/ (accessed 1st January).

Tsou CH (1994). The embryology, reproductive morphology, and systematics of Lecythidaceae. *Memoirs of the New York Botanical Garden* 71:1–110.

Tsou CH, Mori SA (2007). Floral organogenesis and floral evolution of the Lecythidoideae (Lecythidaceae). *Am J Bot* 94:716–736.

Valdes B (1980) A new species of *Pelletiera* (Primulaceae) from Macaronesia. *Candollea* 35:641 - 648.

von Balthazar M, Schönenberger J (2013) Comparative floral structure and systematics in the balsaminoid clade including Balsminaceae, Marcgraviaceae and Tetrameristaceae (Ericales). *Bot J Linn Soc* 173:325-386.

Yang J, Ortega-Hernández J, Gerber S, Butterfield NJ, Hou JB, Lan T, Zhang XG (2015). A superarmored lobopodian from the Cambrian of China and early disparity in the evolution of Onychophora. *Proc Nat Acad Sciences* 112:8678-8683.
