# Supplementary material for: How (much) do flowers vary? Unbalanced disparity among flower functional modules and a mosaic pattern of morphospace occupation in the order Ericales
Source: Proc Biol Sci. 2017 Apr 5;284(1852):20170066. doi: 10.1098/rspb.2017.0066 (PMC5394665; doi:10.1098/rspb.2017.0066)
Supplement: Supplementary figures [file rspb20170066supp2.pdf]

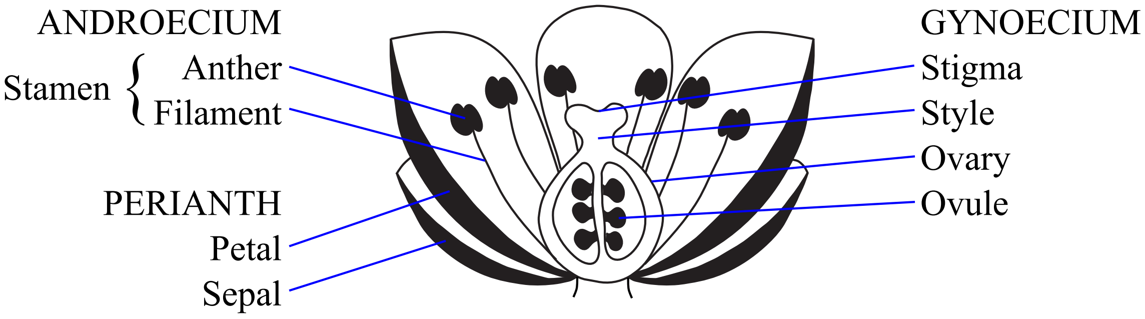

Figure S1. Schematic representation of an angiosperm flower (longitudinal section), showing the different organs of the perianth (sterile part), androecium (male part), and gynoecium (female part).

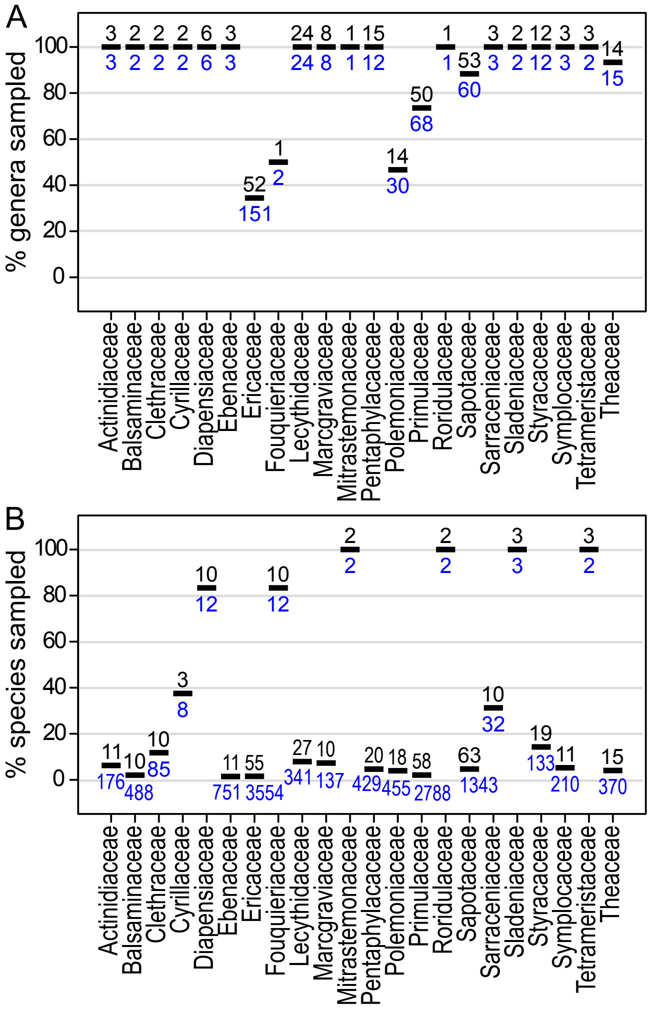

Figure S2. Sample size per family. Black horizontal bars indicate the percentages of species (A) and genera (B) sampled per family relative to the total taxon numbers accepted in The Plant List (The Plant List 2013, http://www.theplantlist.org). For each family, sample size is given in black above each bar and the number of accepted taxa is given in blue below each bar.

Figure S3. 3-dimensionnal PCoA representation of the morphospace of Ericales. Each family and species can be displayed by selecting the corresponding level in the 'Toggle Model Tree' tool ( ) that appears when moving the cursor over the figure in the software Adobe Acrobat. Selecting only the level called 'Ericales' displays the total morphospace and axes. Note that the document might have to be saved on the user's computer to be able to display the 3D components.

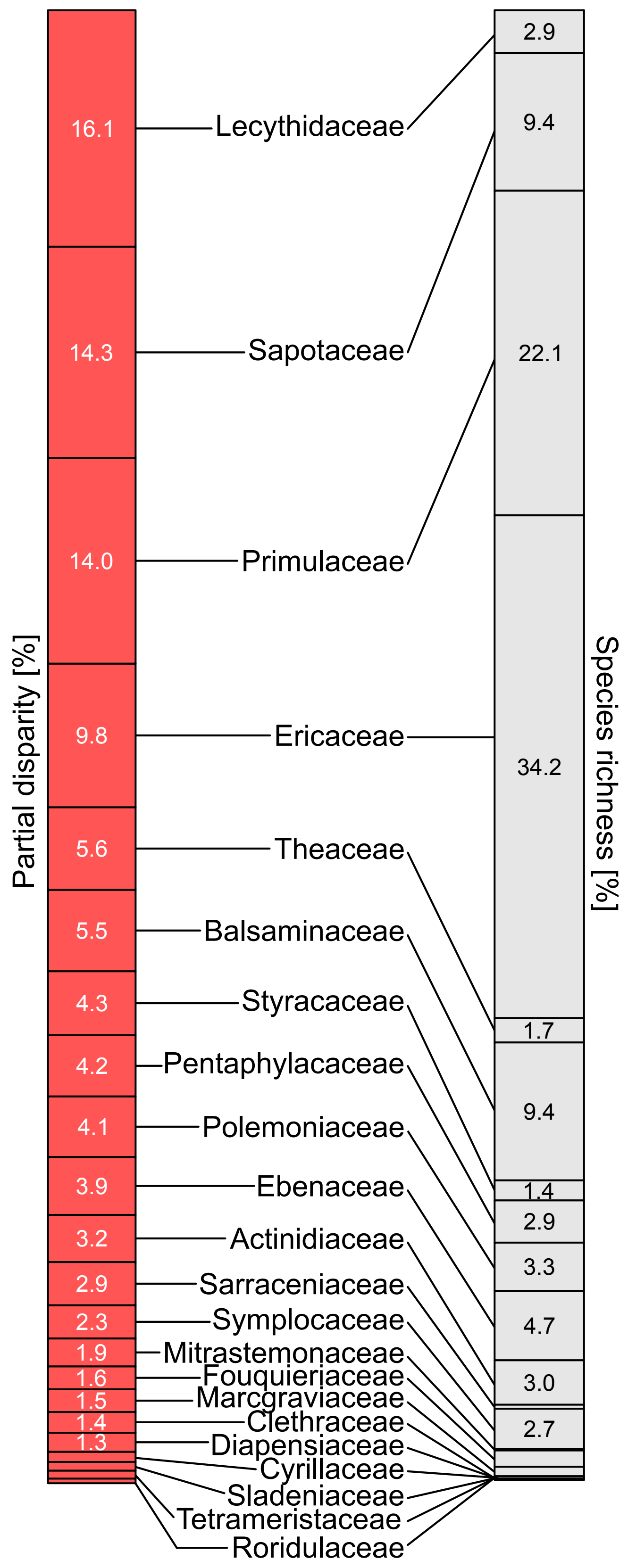

Figure S4. Partial disparity [i.e. additive contribution (in %) of each family to the total disparity] and contribution (in %) to taxonomic diversity for the 22 Ericalean families.

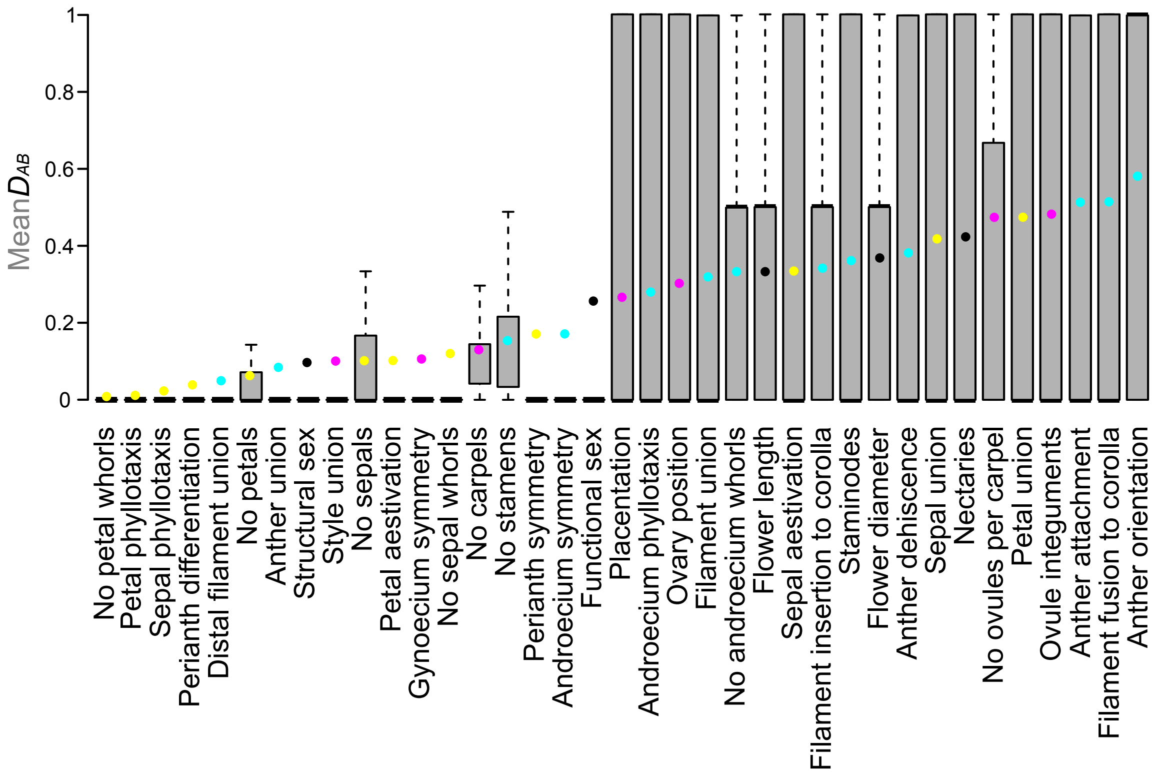

Figure S5. Character variation (*Dchar*) calculated as the mean pairwise difference between taxa for each character. Coloured dots give the value of *D*. Bars indicate standard errors. In yellow: characters describing the perianth, cyan: androecium, magenta: gynoecium, black: general features. The corresponding boxplots (displaying the median d_{AB} for each taxa pair, per character) are displayed in grey.
